# Emergency management: exposure keratopathy

**Published:** 2018-11-09

**Authors:** Wanjiku Mathenge

**Affiliations:** 1 Consultant Ophthalmologist and Director of Training and Research: Rwanda National Institute of Ophthalmology and Dr Agarwal's Eye Hospital, Kigali, Rwanda.

**Exposure keratopathy can result in destruction of the cornea and blindness if not treated urgently.**

Exposure keratopathy (also known as exposure keratitis) is damage to the cornea due to dryness caused by incomplete or inadequate eyelid closure, resulting in loss or insufficiency of the tear film. It is usually a mild condition that is simple to treat. However, it can become an eye emergency in the following situations:

- In unconscious patients in intensive care units, when there is inadequate lid closure
- In patients with a facial nerve palsy, which causes paralysis of the eyelids
- In patients who experience a sudden bleed behind the eye (e.g., after a peribulbar or retrobulbar block)
- In patients who have a condition, such as a tumour, that pushes the eye forward and makes it impossible for the eyelids to close (lagophthalmos)
- Following severe damage to the eyelids (particularly the upper lid), such as trauma, burns or scarring from *Herpes zoster* infection
- If corneal sensation is reduced (e.g., following *Herpes zoster* infection). This makes the eye particularly vulnerable to exposure.

**Protecting unconscious patients**

Reach out to intensive care unit personnel to explain the dangers of exposure and encourage early detection and referral. Prescribe lubricating drops or ointments to all at risk.

If the keratopathy becomes severe, there is a very high risk of irreversible blindness within a matter of hours or days, so treatment must begin immediately.

## Signs and symptoms

In severe cases, the cornea will look dry and may ulcerate, leading to perforation. Patients will experience pain or irritation, foreign body sensation, burning, blurring of vision, watering, redness and sensitivity to light.

## Examination

Assess lid closure and corneal sensitivity. Perform fluorescein staining of the cornea to assess for infection, thinning, scarring or perforation of the cornea.

## Management

Aim to cover, protect and lubricate the cornea. In the case of a sudden bleed, perform a canthotomy (p. 62) to relieve pressure and allow the eyelids to close.

**Figure:**
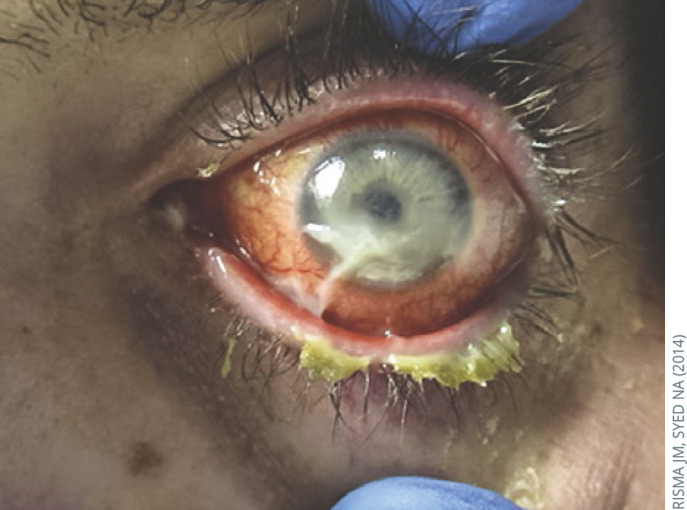
Severe keratopathy with ulceration and keratinisation of the cornea.

In case of proptosis from other causes, take care to protect the cornea from exposure where lid closure is compromised. Prescribe frequent lubricating drops (preservative free if possible to avoid toxicity) during the day and lubricating ointment at night.

In patients who cannot close their eyes, either because they are unconscious, or because of a facial nerve palsy, apply lubricating gel and close the eyelid. This can be as simple as a patch, or lid closure using tape. The lids can also be closed using temporary sutures such as a Frost suture, or partially closed by creating a temporary or permanent tarsorrhaphy.^1^

If the eyelids cannot be closed due to loss of the eyelid or severe proptosis, the cornea can be protected using a moisture chamber. A low-cost moisture chamber can be made using plastic wrap.

## When to refer

Patients with sight-threatening complications such as persistent corneal ulceration, microbial keratitis, perforation and corneal scar should be seen by a specialist. Patients with exposure and reduced corneal sensation should also be referred.
